# Terlipressin-Induced Skin Necrosis in 3 Cases: Should We Concern?

**DOI:** 10.5152/tjg.2024.24408

**Published:** 2024-12-16

**Authors:** Ömer Küçükdemirci, Ahmet Bektaş

**Affiliations:** Department of Gastroenterology and Hepatology, Ondokuz Mayıs University Facultyof Medicine, Samsun, Türkiye

Hepatorenal syndrome with acute kidney injury (HRS-AKI) is a rapidly progressing, often fatal, but potentially reversible decline in renal function. It results from functional hemodynamic decreases in renal arterial perfusion in patients with decompensated cirrhosis. Liver transplantation is the definitive treatment for HRS-AKI. In the absence of liver transplantation, supportive treatment options include the administration of systemic vasoconstrictors, such as somatostatin analogs (octreotide), α-adrenergic agonists, midodrine, and norepinephrine, in conjunction with intravenous (IV) albumin. Terlipressin is a long-acting synthetic vasopressin analog that has been used for over 20 years in Europe for the management of acute bleeding esophageal varices and HRS-AKI, generally presenting fewer complications and less severe side effects compared to vasopressin. The effectiveness of other vasoconstrictors has been studied in several studies; some found them to be less effective than terlipressin, while others reported similar effectiveness. However, recent literature reports have indicated an increasing incidence of acute skin necrosis associated with terlipressin.^1-3^ In this case series, we describe 3 cases from our clinic over the past year where patients developed ischemic skin necrosis while undergoing terlipressin treatment for HRS-AKI.

Three male patients were informed and provided written consent for the use of their medical histories, current findings, and photographs, aged 67 (Case A), 69 (Case B), and 65 (Case C). They had advanced liver cirrhosis due to hepatitis C, hepatitis B, and hepatitis B with hepatocellular carcinoma (HCC), respectively. All 3 patients were on the liver transplant list. Upon presentation, each exhibited a baseline creatinine increase of 50% or more, prompting the discontinuation of all antihypertensive medications, including diuretics and beta-blockers. Despite receiving over 1000 cc of saline infusion within 24 hours, creatinine levels did not regress. Urine outputs progressively decreased, while renal ultrasound findings remained normal. Following nephrology consultation, all 3 patients were diagnosed with HRS-AKI. They were treated with terlipressin at a dosage of 1 mg every 4-6 hours, along with IV albumin at 1 g per kg, with no other vasoactive drugs administered (Table 1).

Following terlipressin treatment, all patients developed ischemic bullae and necrosis, mainly on the lower extremities and scrotum (Case A: Figure 1, Case B: Figure 2, and Case C: Figure 3). None had a prior diagnosis of bullous illness. Notably, Case A had previously received terlipressin for HRS-AKI 3 months earlier without bullae formation. Dermatology specialists confirmed the lesions as ischemic skin necrosis and deemed biopsy unnecessary, leading to terlipressin discontinuation. Subsequent treatment for HRS-AKI continued with saline and supportive care. Cases A and B exhibited significant improvement in skin necrosis within 5-10 days after discontinuation, along with improvement in their HRS-AKI. In Case C, IV norepinephrine was initiated due to refractory hypotension after terlipressin discontinuation. During follow-up, the patient developed anuria and increased creatinine levels. Despite the nephrology team’s initiation of dialysis, Case C died from HRS-AKI complications. However, by the fifth day after stopping terlipressin and starting norepinephrine, regression of skin necrosis was observed.

Terlipressin, a synthetic vasopressin analog, can cause various adverse effects, including abdominal pain, diarrhea, skin discoloration, intestinal and cardiac ischemia, cyanosis, bradycardia, and hypertension. Its vasoconstrictive properties can lead to ischemic necrosis of the skin by acting on V1 receptors, which induce splanchnic vasoconstriction. This reduces portal pressure and enhances glomerular blood flow, but the vasoconstrictive effect also impacts the skin vasculature, resulting in skin necrosis.^4^ Ischemic skin necrosis associated with terlipressin is rare, with approximately 50 cases reported in the literature, showing a male-to-female ratio of about 3 : 1.^3^ Similarly, all our cases involved male patients. Most ischemic skin lesions have been documented in the lower and upper extremities, scrotum, and lumbar region. These lesions generally appear within 24-48 hours of treatment initiation and tend to regress 2-10 days after terlipressin discontinuation.^3^ In our patients treated with terlipressin for HRS-AKI, rapid bullae formation was observed, particularly on the lower extremities and scrotum, with 1 case also affecting the lumbar region, occurring after the 24th hour of treatment without any hemodynamic instability. Within the subsequent 24 hours, these bullae perforated, exposing underlying ecchymotic necrotic areas (see photographs).

Although data are limited, one study indicated that administering terlipressin as an infusion, rather than a bolus, resulted in better treatment success and fewer side effects in managing HRS-AKI.^2^ In our cases, terlipressin was administered as a bolus. While most instances of ischemic skin necrosis in the literature following terlipressin for HRS-AKI were linked to bolus administration, only 2 cases occurred after infusion.^2,3^ However, complications following bolus use remain predominantly reported.^3^ The potential benefits of terlipressin infusion should be further investigated, particularly in patients with recurrent HRS-AKI or acute variceal bleeding who experience complications from bolus therapy.

Discontinuing terlipressin is crucial for managing skin necrosis, as it halts progression and aids healing. Stopping the drug restores blood flow to the affected areas. Supportive care, including wound care with antiseptic ointments, protective dressings, and maintaining cleanliness, is also essential for treatment.^3,5^

There is no information in the literature regarding the continuation of HRS-AKI treatment with less potent vasoconstrictors in patients who develop ischemic skin necrosis due to terlipressin and subsequently have terlipressin discontinued. In our cases, Case A and Case B, following the discontinuation of terlipressin, treatment was continued with IV hydration and albumin, and a complete resolution of ischemic skin lesions was observed. In Case C, however, due to the patient’s need for vasoconstrictor support, norepinephrine treatment was initiated, but the patient ultimately died due to other complications, despite improvement in the signs of ischemic skin necrosis. The strategy of switching to other vasoconstrictors after the discontinuation of terlipressin in patients who develop skin necrosis should be considered in future cases. In the literature, there are no reported cases of patients receiving terlipressin without subsequently developing ischemic skin necrosis. In our series, Case A had received terlipressin treatment for a similar clinical presentation 3 months earlier without any observed side effects. We believe that this adverse effect associated with terlipressin may be more related to the patient’s clinical condition at the time, rather than the dose administered.

Following our first case, our clinical vigilance has increased, potentially leading to the identification of more cases. Patients who receive terlipressin therapy for HRS-AKI are typically in advanced stages of cirrhosis and may receive additional terlipressin treatments. Skin lesions that develop in these patients with cirrhosis could be attributed to alternative causes and may be overlooked. To minimize side effects, the administration of terlipressin as an infusion rather than as a bolus should be investigated. Additionally, switching to other vasoconstrictors should be evaluated in future cases. Clinicians must be vigilant regarding the possibility of terlipressin-induced skin necrosis in this patient group.

## Figures and Tables

**Figure 1.**
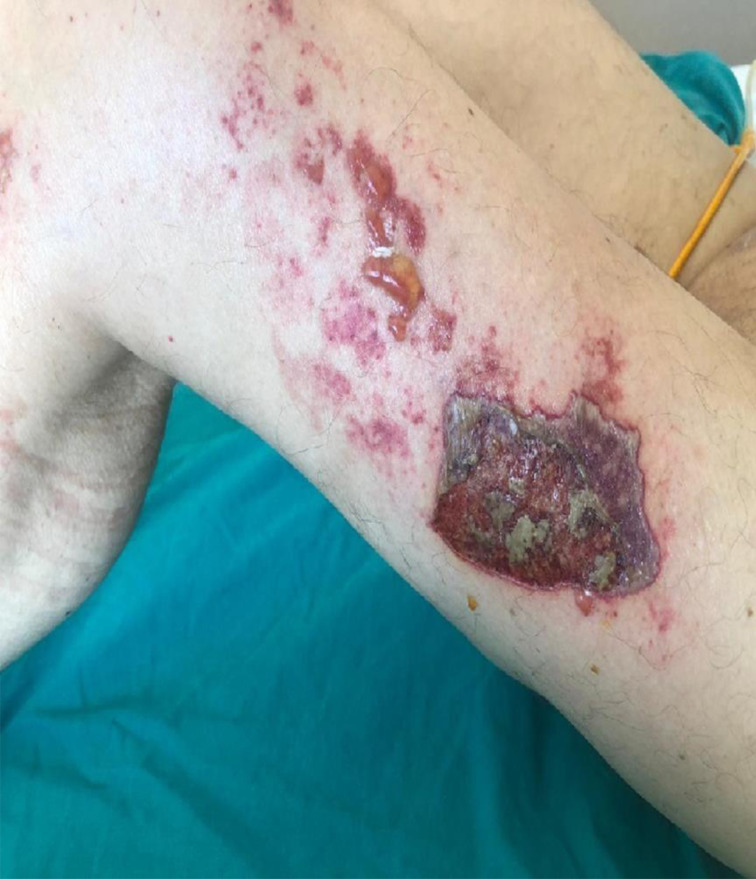
Case A: A 67-year-old male patient with advanced liver cirrhosis due to hepatitis C, presented with an INR of 1.9, total bilirubin of 3 g/dL, creatinine of 1.9 mg/dL, and a calculated MELD score of 24.

**Figure 2.**
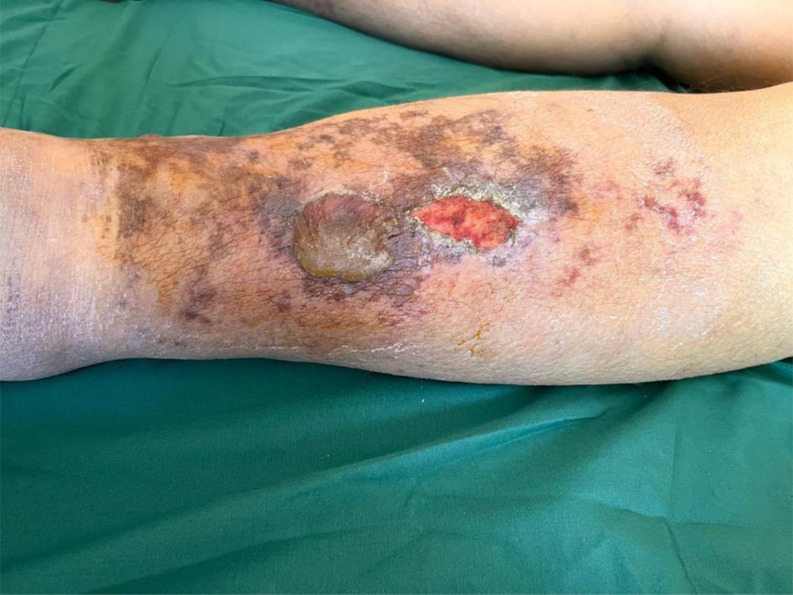
Case B: A 67-year-old male patient with advanced liver cirrhosis due to hepatitis B, presented with an INR of 1.3, total bilirubin of 2.4 g/dL, creatinine of 1.6 mg/dL, and a calculated MELD score of 17.

**Figure 3.**
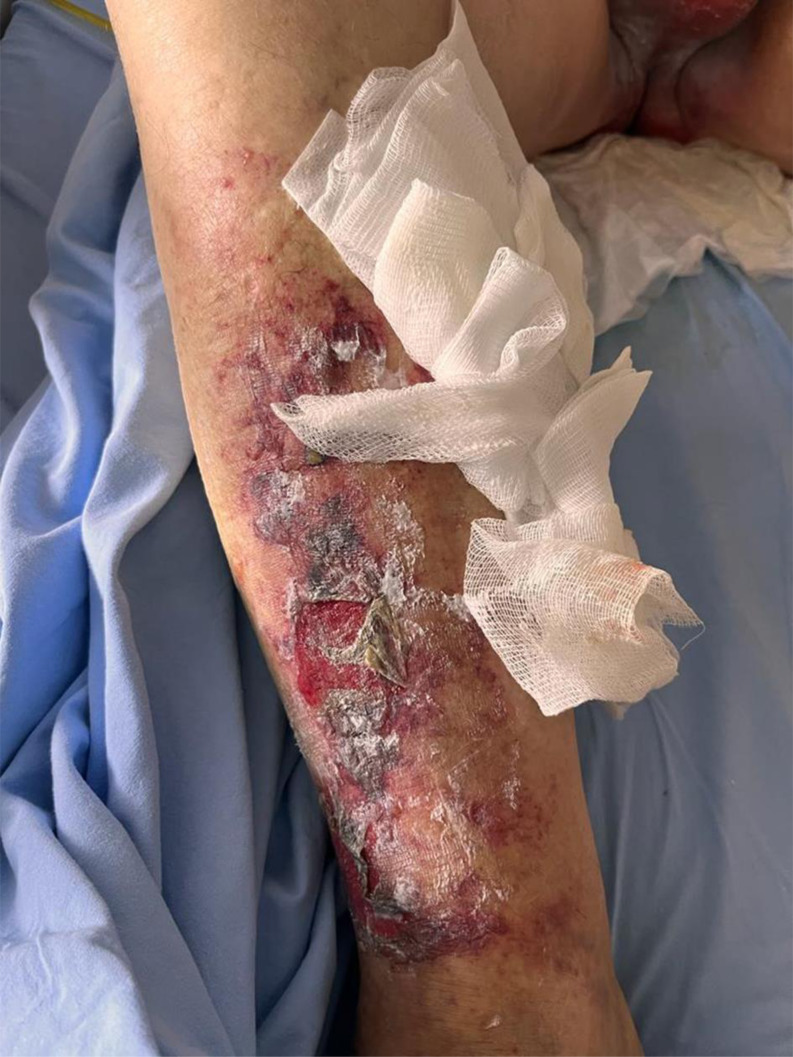
Case C: A 69-year-old male patient with advanced liver cirrhosis and hepatocellular carcinoma due to hepatitis B, presented with an INR of 2.4, total bilirubin of 3.6 g/dL, creatinine of 2.6 mg/dL, and a calculated MELD score of 30.

**Table 1.** Clinical and Laboratory Data for Terlipressin-Induced Skin Necrosis Cases

Characteristic	Case A	Case B	Case C
Age	67	69	65
Gender	Male	Male	Male
Comorbidities	Hepatitis C, liver cirrhosis HBP	Hepatitis B, liver cirrhosis HBP, DM	Hepatitis B, HCC, liver cirrhosis, HBP, CAD
Child–Pugh score	C	C	C
MELD score	24	17	30
Renal ultrasound	No abnormalities	No abnormalities	No abnormalities
INR	1.9	1.3	2.4
Total bilirubin (mg/dL)	3.0	2.4	3.6
Basal creatinine (mg/dL)	0.6	0.7	1.0
Creatinine at administration (mg/dL)	1.9	1.6	2.6
Start day of side effect	24 hours after therapy start	24 hours after therapy start	24 hours after therapy start
Therapy details	Terlipressin (1 mg q 4-6 h), albumin (1 g/kg)	Terlipressin (1 mg q 4-6 h), albumin (1 g/kg)	Terlipressin (1 mg q 4-6 h), albumin (1 g/kg), norepinephrine after discontinuation
Skin necrosis (Location)	Lower extremities, scrotum	Lower extremities, scrotum	Lower extremities, scrotum, lumbar region
Healing of skin lesions after treatment cessation	Significant improvement in 5 days	Significant improvement in 10 days	Improvement by day 5
Outcome	Recovered from HRS-AKI, improved skin necrosis	Recovered from HRS-AKI, improved skin necrosis	Progressed to anuria, death from HRS-AKI complications

CAD, coronary artery disease; DM, diabetes mellitus; HBP, high blood pressure; HRS-AKI, hepatorenal syndrome acute kidney injury; INR, international normalized ratio; MELD, model for end-stage liver disease.

## Data Availability

The data that support the findings of this study are available on request from the corresponding author.
